# Association between enteropathogens and malnutrition in children aged 6–23 mo in Bangladesh: a case-control study^1^,^2^,^3^

**DOI:** 10.3945/ajcn.116.138800

**Published:** 2017-04-05

**Authors:** James A Platts-Mills, Mami Taniuchi, Md Jashim Uddin, Shihab Uddin Sobuz, Mustafa Mahfuz, SM Abdul Gaffar, Dinesh Mondal, Md Iqbal Hossain, M Munirul Islam, AM Shamsir Ahmed, William A Petri, Rashidul Haque, Eric R Houpt, Tahmeed Ahmed

**Affiliations:** 4 Division of Infectious Diseases and International Health, University of Virginia, Charlottesville, VA; and; 5 International Center for Diarrhoeal Disease Research, Bangladesh, Dhaka, Bangladesh

**Keywords:** children, enteropathogens, malnutrition, PCR, low-resource settings, diarrhea

## Abstract

**Background:** Early exposure to enteropathogens has been associated with malnutrition in children in low-resource settings. However, the contribution of individual enteropathogens remains poorly defined. Molecular diagnostics offer an increase in sensitivity for detecting enteropathogens but have not been comprehensively applied to studies of malnutrition.

**Objective:** We sought to identify enteropathogens associated with malnutrition in Bangladesh.

**Design:** Malnourished children [weight-for-age *z* score (WAZ) <−2] aged 6–23 mo in Dhaka, Bangladesh, and identified by active community surveillance were enrolled as cases, and normal-weight children (WAZ >−1) of the same age and from the same community were enrolled as controls. Stools were collected at enrollment and, for cases, after a 5-mo nutritional intervention. Enrollment and follow-up stools were tested by quantitative polymerase chain reaction for 32 enteropathogens with the use of a custom-developed TaqMan Array Card.

**Results:** Enteropathogen testing was performed on 486 cases and 442 controls upon enrollment and 365 cases at follow-up. At enrollment, the detection of enteroaggregative *Escherichia coli* (OR: 1.39; 95% CI: 1.05, 1.83), *Campylobacter* spp. (OR: 1.46; 95% CI: 1.11, 1.91), heat-labile enterotoxin-producing *E. coli* (OR: 1.55; 95% CI: 1.04, 2.33), *Shigella*/enteroinvasive *E. coli* (OR: 1.65; 95% CI: 1.10, 2.46), norovirus genogroup I (OR: 1.66; 95% CI: 1.23, 2.25), and *Giardia* (OR: 1.73; 95% CI: 1.20, 2.49) were associated with malnourished cases, and the total burden of these pathogens remained associated with malnutrition after adjusting for sociodemographic factors. The number of these pathogens at follow-up was negatively associated with the change in WAZ during the intervention (−0.10 change in WAZ per pathogen detected; 95% CI: −0.14, −0.06), whereas the number at enrollment was positively associated with the change in WAZ (0.05 change in WAZ per pathogen detected; 95% CI: 0.00, 0.10).

**Conclusions:** A subset of enteropathogens was associated with malnutrition in this setting. Broad interventions designed to reduce the burden of infection with these pathogens are needed. This trial was registered at clinicaltrials.gov as NCT02441426.

## INTRODUCTION

Child undernutrition remains an important risk factor for both mortality and impaired long-term development in low-resource settings (1, 2). Early exposure to enteropathogens has been associated with poor child growth in these settings; however, the role of specific enteropathogens has not been comprehensively evaluated. Most prior studies have evaluated a limited number of pathogens and have revealed associations with *Shigella* and heat-labile enterotoxin-producing *Escherichia coli* (LT-ETEC)^7^ (3, 4), enteroaggregative *E. coli* (EAEC) (5), *Campylobacter* (4, 6), *Cryptosporidium* (7–9), *Giardia* (10–12), and *Ascaris* (13). Furthermore, interventional studies designed to reduce enteropathogen exposure have primarily used diarrhea and growth as outcomes because, to our knowledge, appropriate enteropathogen-specific outcomes have not been established.

The development of highly sensitive molecular diagnostics for a wide range of enteropathogens has provided new insight into the etiology of diarrhea in children in low-resource settings (14–16). In this study (NCT02441426), we sought to use a broad molecular diagnostic approach to identify enteropathogens associated with malnutrition in a case-control study of children aged 6–23 mo in Dhaka, Bangladesh.

## METHODS

### Study design and sample collection

The study was conducted in Mirpur, a subdistrict of Dhaka, Bangladesh. The study site has been described in detail (17), and the results of the nutritional intervention have been reported elsewhere (18). Briefly, children aged 6–23 mo presenting to a community malnutrition clinic with a weight-for-age *z* score (WAZ) <−2 were eligible for inclusion as a case. Children were eligible if they had diarrheal symptoms upon presentation; however, children with diarrhea that was severe (defined as needing intravenuous hydration or hospitalization) or persistent (defined as a duration >14 d) were excluded. Contemporaneous controls, frequency matched by age, sex, and area of residence, and a WAZ >−1 were actively enrolled from the same neighborhood (the Bauniabadh area of Mirpur). Enrollment was performed from November 2009 to December 2012. After enrollment, cases were given 2 daily macronutrient supplements [Pushti packets (18, 19)] for 5 mo or until reaching a WAZ >−1; severely malnourished cases (WAZ <−3) were given an additional packet each day. Both cases and controls received a micronutrient powder (MoniMix; Renata Limited) for ≥2 mo (children enrolled after August 2010 were given the micronutrient powder for 4 mo). The contents of both supplements are described in **Supplemental Table 1**. All children aged ≥1 y were given 200 mg albendazole at enrollment, and all diarrheal episodes at enrollment and during follow-up were treated with an oral rehydration solution and zinc. Weights were obtained both at enrollment and after the completion of the intervention with the use of digital scales. Data on household food insecurity, income, water access and treatment, personal hygiene, latrine access, and feeding practices were obtained at enrollment via a maternal questionnaire. Insufficient food in the home was defined as a maternal report of concern that there was not enough food in the home during the month before enrollment. The age of cessation of exclusive breastfeeding was defined as the age in months at which the mother reported sustained introduction of nonbreastmilk liquid or food into the diet. Approval was obtained from the institutional ethics review boards at the International Center for Diarrheal Disease Research, Bangladesh, and the University of Virginia.

### Stool testing

A quantitative polymerase chain reaction (qPCR) of stools was performed with the use of a custom-developed TaqMan Array Card that compartmentalized probe-based real-time PCR assays for 32 enteropathogens. This was performed in 2015 on all available archived stools from the enrollment as well as from cases after the completion of the 5-mo intervention. First, nucleic acid was extracted from specimens with the QIAamp Fast DNA Stool mini kit (Qiagen). Two external controls, MS2 bacteriophage and Phocine herpesvirus, were added to the stools during extraction to confirm nucleic acid extraction and amplification efficiency. The analytic cutoff of each pathogen was a quantification cycle (*C_q_*) of 35; thus, a *C_q_* > 35 was considered negative, as described previously (14). Pathogen quantities were reported on a log scale on which each *C_q_* value decrease represented a 2-fold increase in pathogen quantity. Enrollment stools were also tested for bacteria by culture and protozoa with the use of an enzyme immunoassay (EIA) as described previously (20). Specifically, EIA was performed for *Campylobacter* (ProSpecT) and *E. histolytica*, *Giardia*, and *Cryptosporidium* (TechLab). Testing for diarrheagenic *E. coli* was performed by pooling 5 lactose-fermenting colonies for multiplex PCR for shiga toxin 1 (*stx1*), shiga toxin 2 (*stx2*), heat-stabile enterotoxin (*ST*), heat-labile enterotoxin (*LT*), intimin (*eae*), bundle-forming pilus (*bfpA*), invasion plasmid antigen H (*ipaH*), AggR-activated Transporter (gene A) (*aatA*), and AggR-activated Island (gene C) (*aaiC*).

### Statistical analysis

We used univariable logistic regression to describe the association between sociodemographic factors and case-control status. To identify relations between enteropathogen detection and case-control status, we fit a logistic regression model by dichotomously detecting pathogens by either the original microbiological workup or PCR and adjusting for age at enrollment, sex, and diarrhea at enrollment. To estimate the association between the number of malnutrition-associated pathogens detected and case-control status, we fit a logistic regression model, adjusting for age at enrollment, sex, and diarrhea at enrollment, as well as sociodemographic factors of interest identified by regression as described previously.

To describe the association between pathogen presence at enrollment and follow-up and the change in WAZ in cases, we fit a multivariable linear regression model for each pathogen, with the change in WAZ from enrollment until the end of the intervention as the outcome and the baseline WAZ, sex, enrollment age, sociodemographic factors, and presence of the pathogen at both enrollment and follow-up as predictors. All statistical analyses were performed with the use of R version 3.2.2 (R Foundation for Statistical Computing).

## RESULTS

**FIGURE 1:**
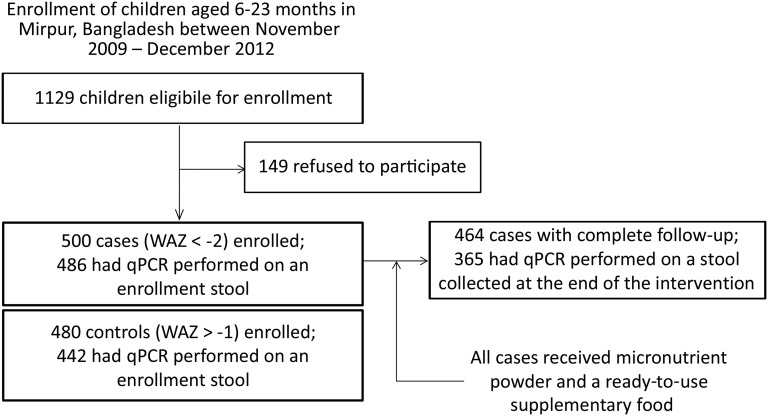
Study flow diagram. qPCR, quantitative polymerase chain reaction; WAZ, weight-for-age *z* score.

A total of 500 cases and 480 controls were enrolled, of whom 95% (928/980) had an enrollment stool available, and 73% (365/500) of cases had both repeat anthropometry and qPCR performed on a follow-up stool (**Figure 1**). First, we identified sociodemographic characteristics at enrollment associated with malnutrition (**Table 1**). Cases were slightly older than controls, were breastfed for a shorter duration, and came from families with a lower monthly income. Mothers of case children were more likely to describe food insecurity and have a primary drinking water source outside the home than those of control children and were less likely to routinely treat their drinking water. Malnourished children were not more likely to have diarrhea at the time of enrollment. The prevalence of stunting and wasting was strikingly higher in cases.

**TABLE 1:** Study population characteristics^1^

Sociodemographic factors	Cases(*n* = 486)^2^	Controls (*n* = 442)^3^	ORs (95% CIs)	*P* values
Age, mo	14 (9–19)^4^	11 (8–15)	1.09 (1.06, 1.11)	<0.001
Females, *n* (%)	232 (47.7)	218 (49.3)	0.94 (0.73, 1.21)	0.630
Wasted at enrollment (WLZ <−2), *n* (%)	187 (38.5)	1 (0.2)	275.81 (38.48, 1976.97)	<0.001
Stunted an enrollment (LAZ <−2), *n* (%)	342 (70.4)	22 (5.0)	45.34 (28.31, 72.60)	<0.001
Diarrhea at enrollment, *n* (%)	14 (2.9)	22 (5.0)	0.57 (0.29, 1.12)	0.103
Insufficient food in the home, *n* (%)	228 (46.9)	154 (34.8)	1.65 (1.27, 2.15)	<0.001
Monthly income (thousand taka), *n* (%)	7 (5–9)	8 (6–12)	0.92 (0.90, 0.95)	<0.001
Primary drinking water source outside the home, *n* (%)	443 (91.2)	356 (80.5)	2.49 (1.68, 3.68)	<0.001
Routine treatment of drinking water, *n* (%)	292 (60.1)	322 (72.9)	0.56 (0.43, 0.74)	<0.001
Handwashing after using toilet, *n* (%)	129 (26.5)	94 (21.3)	1.34 (0.99, 1.81)	0.061
Access to flushing toilet, *n* (%)	162 (33.3)	157 (35.5)	0.91 (0.69, 1.19)	0.484
Current exclusive or partial breastfeeding, *n* (%)	452 (93.0)	416 (94.1)	0.83 (0.49, 1.41)	0.491
Age of cessation of exclusive breastfeeding, mo	3 (0–5)	4 (0–6)	0.96 (0.91, 1.01)	0.154

1 Univariate logistic regression was used to estimate the ORs (95% CIs) and *P* values. LAZ, length-for-age *z* score; WAZ, weight-for-age *z* score; WLZ, weight-for-length *z* score.

2 WAZ <−2.

3 WAZ >−1.

4 Median; IQR in parentheses (all such values).

**FIGURE 2:**
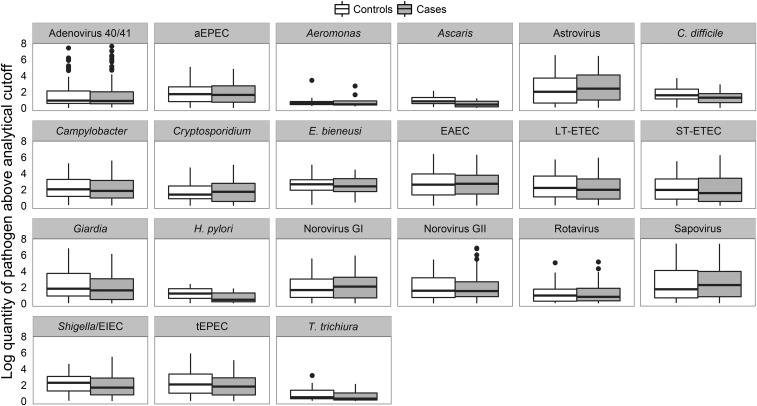
Pathogen quantity when detected with the use of quantitative polymerase chain reaction in cases and controls. Box-and-whisker plots are shown with the use of Tukey’s method, in which the bottom and top of the box represent the first and third quartiles, respectively, the line within the box represents the median, the whiskers extend from the box to all values within 1.5 times the IQR, and points beyond the whiskers represent outliers. For all pathogens, there was no statistically significant difference in the quantity between cases and controls (Mann-Whitney test; *P* > 0.05). aEPEC, atypical enteropathogenic *E. coli*; EAEC, enteroaggregative *E. coli*; EIEC, enteroinvasive *E. coli*; GI, genogroup I; GII, genogroup II; LT-ETEC, heat-labile enterotoxin-producing *E. coli*; ST-ETEC, heat-stable enterotoxin-producing *E. coli*; tEPEC, typical enteropathogenic *E. coli*.

qPCR detection of EAEC (OR: 1.39; 95% CI: 1.05, 1.83), *Campylobacter* spp. (OR: 1.46; 95% CI: 1.11, 1.91), LT-ETEC (OR: 1.55; 95% CI: 1.04, 2.33), *Shigella*/enteroinvasive *E. coli* (EIEC) (OR: 1.65; 95% CI: 1.10, 2.46), norovirus genogroup II (OR: 1.66; 95% CI: 1.23, 2.25), and *Giardia* (OR: 1.73; 95% CI: 1.20, 2.49) was associated with malnutrition at enrollment after adjusting for enrollment age, sex, and the presence of diarrhea at enrollment (**Table 2**). After further adjusting for sociodemographic factors, the association remained statistically significant for EAEC, norovirus genogroup II, and *Giardia*. Mixed infection with these malnutrition-associated pathogens was common (mean ± SD for cases: 1.86 ± 0.93; mean ± SD for controls: 1.45 ± 0.97; Mann-Whitney test; *P* < 0.001), and the detection of these pathogens was associated with malnutrition in a dose-dependent fashion, an association that persisted after adjusting for sociodemographic factors (**Table 3**). For all pathogens, there was no statistically significant difference in the quantity detected between cases and controls (**Figure 2**). Culture- and EIA-based diagnostics did not reveal any statistically significant associations (**Supplemental Table 2**). Similar associations as those between qPCR pathogen detection and low WAZ were observed between qPCR pathogen detection and stunted compared with nonstunted children (**Supplemental Table 3**).

**TABLE 2:** Pathogen detection by TaqMan Array Card in cases and controls^1^

			Crude^2^		Adjusted^3^	
	Cases(*n* = 486)	Controls(*n* = 442)	ORs(95% CIs)	*P* values	ORs(95% CIs)	*P* values
Bacteria, *n* (%)						
*Aeromonas* spp.	8 (1.6)	9 (2.0)	0.92 (0.34, 2.48)	0.874	0.77 (0.28, 2.11)	0.606
*Campylobacter jejuni/coli*	149 (30.7)	101 (22.9)	1.46 (1.08, 1.97)	0.014	1.26 (0.92, 1.72)	0.149
*Campylobacter* spp.	218 (44.9)	153 (34.6)	1.46 (1.11, 1.91)	0.007	1.22 (0.92, 1.63)	0.165
*Clostridium difficile*	18 (3.7)	15 (3.4)	1.36 (0.66, 2.78)	0.401	1.57 (0.74, 3.32)	0.237
EAEC	311 (64)	264 (59.7)	1.39 (1.05, 1.83)	0.020	1.36 (1.02, 1.82)	0.035
aEPEC	128 (26.3)	118 (26.7)	0.92 (0.68, 1.24)	0.580	0.95 (0.69, 1.30)	0.744
tEPEC	83 (17.1)	79 (17.9)	1.01 (0.71, 1.42)	0.967	0.93 (0.65, 1.34)	0.699
LT-ETEC	73 (15.0)	45 (10.2)	1.55 (1.04, 2.33)	0.033	1.43 (0.94, 2.17)	0.098
ST-ETEC	141 (29)	105 (23.8)	1.26 (0.94, 1.71)	0.127	1.27 (0.93, 1.75)	0.135
*Shigella*/EIEC	93 (19.1)	46 (10.4)	1.65 (1.10, 2.46)	0.014	1.47 (0.97, 2.23)	0.070
Viruses, *n* (%)						
Adenovirus 40/41	99 (20.4)	86 (19.5)	1.14 (0.82, 1.58)	0.451	1.18 (0.83, 1.66)	0.358
Astrovirus	46 (9.5)	40 (9.0)	1.10 (0.70, 1.73)	0.681	1.03 (0.64, 1.63)	0.912
Norovirus GI	50 (10.3)	35 (7.9)	1.31 (0.83, 2.09)	0.247	1.37 (0.84, 2.22)	0.204
Norovirus GII	148 (30.5)	98 (22.2)	1.66 (1.23, 2.25)	0.001	1.73 (1.26, 2.37)	0.001
Rotavirus	48 (9.9)	52 (11.8)	0.99 (0.65, 1.52)	0.976	1.07 (0.69, 1.67)	0.758
Sapovirus	149 (30.7)	128 (29.0)	1.09 (0.82, 1.45)	0.567	1.07 (0.79, 1.44)	0.680
Parasites, *n* (%)						
*Ascaris lumbricoides*	14 (2.9)	5 (1.1)	1.88 (0.66, 5.36)	0.239	1.79 (0.61, 5.31)	0.292
*Cryptosporidium*	44 (9.1)	39 (8.8)	0.93 (0.59, 1.48)	0.768	0.89 (0.55, 1.45)	0.651
*Enterocytozoon bieneusi*	54 (11.1)	34 (7.7)	1.20 (0.76, 1.91)	0.433	1.00 (0.62, 1.62)	0.998
*Giardia*	109 (22.4)	55 (12.4)	1.73 (1.20, 2.49)	0.003	1.51 (1.04, 2.20)	0.031
*Trichuris trichiura*	21 (4.3)	14 (3.2)	0.99 (0.49, 2.01)	0.981	0.90 (0.43, 1.89)	0.787

1 All pathogens detected in ≥1% of stools are shown. aEPEC, atypical enteropathogenic *E. coli*; EAEC, enteroaggregative *E. coli*; EIEC, enteroinvasive *E. coli*; GI, genogroup I; GII, genogroup II; LT-ETEC, heat-labile enterotoxin-producing *E. coli*; ST-ETEC, heat-stable enterotoxin-producing *E. coli*; tEPEC, typical enteropathogenic *E. coli*.

2 Estimated with the use of multivariable logistic regression and adjusted for enrollment age, sex, and diarrhea at enrollment.

3 Estimated with the use of multivariable logistic regression and adjusted for enrollment age, sex, diarrhea at enrollment, insufficient food in the home, income, location of primary water source, routine treatment of drinking water, and age of cessation of exclusive breastfeeding.

**TABLE 3:** Adjusted association between the number of malnutrition-associated pathogens detected and a WAZ <−2^1^

			Crude^3^		Adjusted^4^	
Pathogens detected^2^	Cases (*n* = 486)	Controls (*n* = 442)	ORs (95% CIs)	*P* values	ORs (95% CIs)	*P* values
0	40 (8.2)	79 (17.9)	Reference	—	Reference	—
1	130 (26.7)	159 (36.0)	1.70 (1.08, 2.69)	0.023	1.55 (0.96, 2.49)	0.073
2	177 (36.4)	131 (29.6)	2.70 (1.71, 4.26)	<0.001	2.32 (1.44, 3.72)	<0.001
≥3	139 (28.6)	73 (16.5)	3.82 (2.35, 6.22)	<0.001	3.02 (1.82, 5.01)	<0.001

1 WAZ, weight-for-age *z* score.

2 Defined as the number of detections of *Campylobacter*, enteroaggregative *E. coli*, heat-labile enterotoxin-producing *E. coli*, *Shigella*/enteroinvasive *E. coli*, norovirus genogroup II, and *Giardia* with the use of polymerase chain reaction from the stool collected at enrollment.

3 Estimated with the use of multivariable logistic regression and adjusted for enrollment age, sex, and diarrhea at enrollment.

4 Estimated with the use of multivariable logistic regression and adjusted for enrollment age, sex, diarrhea at enrollment, insufficient food in the home, income, location of primary water source, routine treatment of drinking water, and age of cessation of exclusive breastfeeding.

**FIGURE 3:**
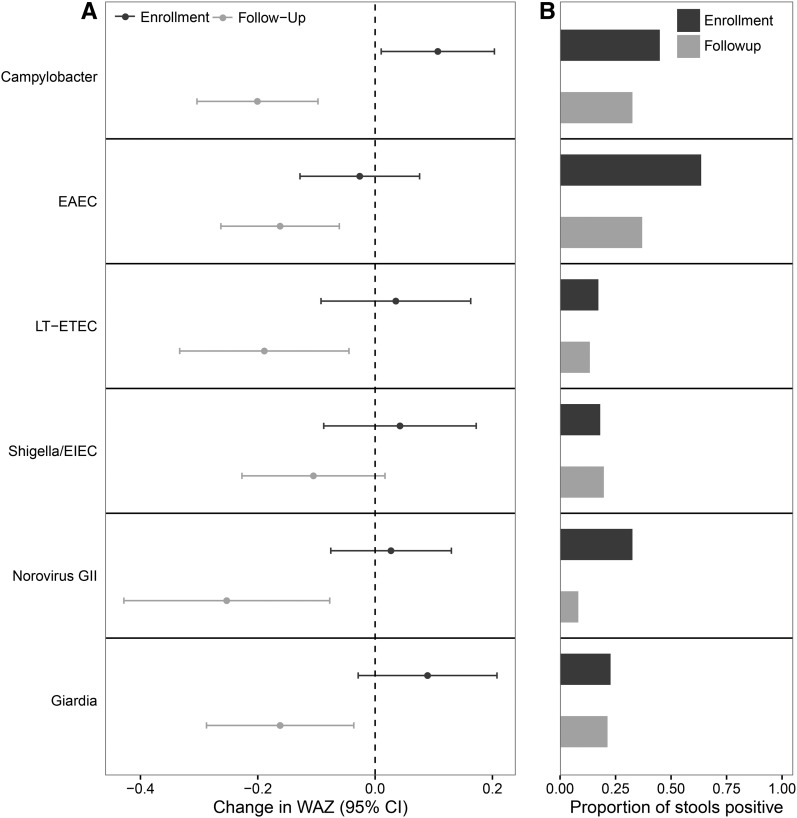
Association between pathogen detection at both enrollment and follow-up and change in weight during follow-up derived from a multivariable linear regression model for each pathogen, with the change in WAZ from enrollment until the end of the intervention as the outcome and the baseline WAZ, enrollment age, sex, diarrhea at enrollment, insufficient food in the home, income, location of primary water source, routine treatment of drinking water, and age of cessation of exclusive breastfeeding as well as the presence of the pathogen at both enrollment and follow-up as predictors. The *x* axis in panel A shows the difference in the change in WAZ from enrollment to the completion of a 5-mo nutritional intervention for cases in which each pathogen was detected compared with not being detected at enrollment (black) and after the completion of the intervention (gray); panel B shows the proportion of stools in which these pathogens were detected at enrollment (black) and follow-up (gray). EAEC, enteroaggregative *E. coli*; EIEC, enteroinvasive *E. coli*; GII, genogroup II; LT-ETEC, heat-labile enterotoxin-producing *E. coli*; WAZ, weight-for-age *z* score.

The 5-mo nutritional intervention did not substantially increase the WAZ of the malnourished cases (mean ± SD change in WAZ: 0.09 ± 0.50). For most pathogens, there was no correlation between detection at enrollment and follow-up in the same individual, with the exception of *Campylobacter* (OR: 1.70; 95% CI: 1.07, 2.71; *P* = 0.019), *C. difficile* (OR: 11.34; 95% CI: 1.00, 74.89; *P* = 0.025), *Giardia* (OR: 3.46; 95% CI: 1.94, 6.18; *P* < 0.001), and *Trichuris* (OR: 14.08; 95% CI: 3.58, 50.65; *P* < 0.001) (all Fisher’s exact tests). We then examined the enrollment stools of cases to identify whether these pathogens were associated with the change in WAZ during follow-up (**Figure 3**). Although children with *Campylobacter*, EAEC, LT-ETEC, *Shigella*/EIEC, norovirus genogroup II, and *Giardia* were more likely to be malnourished, the detection of these pathogens at enrollment was not associated with a lower change in WAZ during follow-up. In fact, the detection of *Campylobacter*, as well as the total number of these pathogens detected at enrollment (0.05 change in WAZ per pathogen detected; 95% CI: 0.00, 0.10; *P* = 0.040), were marginally positively associated with the change in WAZ during the follow-up period.

Finally, we examined follow-up stools from cases to identify pathogens associated with a lower change in WAZ over the 5-mo follow-up period (Figure 3). The detection of norovirus genogroup II, LT-ETEC, *Giardia*, EAEC, and *Campylobacter* were again associated with a lower change in WAZ during follow-up, whereas *Shigella*/EIEC detection was not associated with the change in WAZ. The total number of these pathogens detected in follow-up stools was also negatively associated with the change in WAZ (−0.10 change in WAZ per pathogen detected; 95% CI: −0.14, −0.06; *P* < 0.001).

## DISCUSSION

In this case-control study of malnourished children aged <2 y in Dhaka, Bangladesh, molecular diagnostics increased the sensitivity and breadth of enteropathogen detection and identified associations between enteropathogen infection and malnutrition. Infection with *Campylobacter*, EAEC, LT-ETEC, *Shigella*/EIEC, norovirus genogroup II, and *Giardia* was associated with malnourished cases (defined herein as WAZ <−2). Furthermore, infection with these malnutrition-associated pathogens remained strongly associated with malnutrition after controlling for potential confounding by sociodemographic factors. The effect sizes were clearly reduced by adjusting for these factors, suggesting that they are independently associated with both enteropathogen infection and malnutrition. For each individual pathogen, there was no significant association between quantitative detection and malnourished cases. We presume that this is because with high quantities of most of these pathogens diarrhea becomes likely (14), and children with severe and persistent diarrhea were excluded, whereas less severe diarrhea was rare at enrollment in this study. That is, the detection of high quantities of these pathogens in the absence of diarrhea is uncommon. We did, however, observe that the detection of these pathogens was associated with malnutrition. This observation supports the link between these enteropathogen infections and malnutrition in these settings. Plausible mechanisms exist for the relation between many of these pathogens and malnutrition, including increases in intestinal inflammation and permeability, that are characteristic of environmental enteric dysfunction (21, 22).

The association with malnutrition was predominantly seen for bacterial enteropathogens, some of which were highly prevalent in these children. *Shigella*, *Campylobacter*, and EAEC are invasive enteropathogens that have clearly been associated with inflammation and gut barrier disruption (5, 23, 24). Enterotoxigenic *E. coli* has also been associated with poor growth in several studies (3, 4), and it has been postulated that heat-labile toxin serves as a marker of the presence of certain colonization factors that may mediate the association with malnutrition (25). The recent application of culture-independent diagnostic tests for *Campylobacter* has revealed a substantially higher prevalence than previously appreciated, with a substantial burden of disease (26). The strength of the association with cases was similar for detecting *Campylobacter jejuni/coli* as well the *Campylobacter* genus in general. Additional work is needed to identify the burden and impact of diverse *Campylobacter* spp. in these settings (27). *Giardia* has also been associated with poor growth (11). The finding of an association between norovirus infection and malnutrition is new to our knowledge. Immune dysfunction is a risk factor for norovirus infection and is associated with persistent shedding of this organism (28–30). One could speculate that the increased detection of norovirus in cases may be a sequela of impaired mucosal immunity. A strength of this work is the testing for a broad range of pathogens so that prevalence and effect sizes could be compared side by side. It is striking that multiple pathogens were associated with malnutrition, and that infection with many of these pathogens was common. Interventions aimed to broadly reduce the exposure to multiple pathogens will be needed to affect growth improvements.

Although malnourished cases were more likely to have infections with these pathogens than were normal-weight controls, enteropathogen infection at enrollment in cases did not predict lower weight gain over the course of the intervention. Indeed, the detection of *Campylobacter* and a higher pathogen burden at enrollment were associated with a higher change in WAZ during follow-up. This supports the notion that enteric infections can cause weight loss but that recovery is possible. Meanwhile, infections after enrollment and thus detected at follow-up were associated with a lower change in WAZ, suggesting cycles of subclinical enteropathogen infection with negative impacts on weight and interludes of catch-up growth, as has been clearly described with overt diarrhea (31). *Campylobacter* and *Giardia* detection upon enrollment was associated with detection at follow-up, suggesting either that these pathogens may be persistently carried for months or that repeated exposure is common, or both. If the former is the case, then targeted treatment interventions for these pathogens may be more likely to have an enduring effect. Higher-resolution prospective studies with both frequent stool collection and genotyping are needed to distinguish reinfection from persistence with these pathogens.

Studies of environmental interventions, including water treatment, the promotion of exclusive breastfeeding, and improved sanitation and hygiene have generally used diarrhea and occasionally linear and ponderal growth as the primary outcomes for assessing the efficacy of the intervention. However, it has been proposed that enteropathogen infection may be a more proximal outcome measure (32). In this study, we identified a subset of enteropathogens that are associated with malnutrition and thus might form the basis for such an outcome. Validating a single metric of enteropathogen burden would best be performed in a prospective cohort study.

This study has several limitations. First, the definition of malnutrition (WAZ <−2) used in this study, although widely endorsed, is broad and may limit the specificity of the identified associations. Second, a case-control study does not allow for an elucidation of the temporal relation between enteropathogen infection and malnutrition. Malnutrition has a well-established association with immunosuppression (33), and thus the increase in enteropathogen carriage seen herein may be a sequela of malnutrition rather than a cause. However, a case-control design is an efficient way to broadly identify pathogens that are associated with malnutrition in these children. In addition, this was an exploratory analysis of a broad range of pathogens designed to broadly screen for pathogens that may be associated with malnutrition. These findings should be confirmed in subsequent studies that ideally would allow for some causal inference as to the role of these pathogens in the development of malnutrition. Finally, these findings may not be generalizable to other settings, although many of the pathogens identified herein have been associated with malnutrition in other studies.

In summary, screening for enteropathogens directly from stool specimens with highly sensitive molecular assays revealed associations between several prevalent enteropathogens and malnutrition. The association between the burden of these enteropathogens and malnutrition persisted after adjusting for sociodemographic factors. This study provides a list of specific pathogens that are putative contributors to poor growth and development in children in low-resource settings and provides a potential target for future interventions.

## Footnotes

7 Abbreviations used: *C_q_*, quantification cycle; EAEC, enteroaggregative *E. coli*; EIA, enzyme immunoassay; EIEC, enteroinvasive *E. coli*; LT-ETEC, heat-labile enterotoxin-producing *E. coli*; PCR, polymerase chain reaction; qPCR, quantitative polymerase chain reaction; WAZ, weight-for-age *z* score.
